# Anti-inflammatory effect of Tauroursodeoxycholic acid in RAW 264.7 macrophages, Bone marrow-derived macrophages, BV2 microglial cells, and spinal cord injury

**DOI:** 10.1038/s41598-018-21621-5

**Published:** 2018-02-16

**Authors:** Seong Jun Kim, Wan-Kyu Ko, Min-Jae Jo, Yoshie Arai, Hyemin Choi, Hemant Kumar, In-Bo Han, Seil Sohn

**Affiliations:** 1 Department of Neurosurgery, CHA University, CHA Bundang Medical Center, Seongnam-si, Gyeonggi-do Republic of Korea; 2 Department of Biomedical Science, CHA University, Seongnam-si, Gyeonggi-do Republic of Korea

## Abstract

This study aimed to investigate the anti-inflammatory effects of tauroursodeoxycholic acid (TUDCA) after spinal cord injury (SCI) in rats. We induced an inflammatory process in RAW 264.7 macrophages, BV2 microglial cells, and bone marrow-derived macrophages (BMM) using lipopolysaccharide (LPS). The anti-inflammatory effects of TUDCA on LPS-stimulated RAW 264.7 macrophages, BV2 microglial cells, and BMMs were analyzed using nitric oxide (NO) assays, quantitative real-time polymerase chain reactions (qRT-PCR), and enzyme-linked immunosorbent assays (ELISA). The pathological changes in lesions of the spinal cord tissue were evaluated by hematoxylin & eosin (H&E) staining, luxol fast blue/cresyl violet-staining and immunofluorescent staining. TUDCA decreased the LPS-stimulated inflammatory mediator, NO. It also suppressed pro-inflammatory cytokines of tumor necrosis factor-α (TNF-α), interleukin 1-β (IL-1β), cyclooxygenase-2 (COX-2), and inducible nitric oxide synthase (iNOS) in both mRNA and protein levels. In addition, TUDCA decreased prostaglandin E_2_ (PGE_2_). After SCI, TUDCA supported the recovery of the injury site and suppressed the expression of inflammatory cytokines such as iNOS, CD68 and CD86. In addition, TUDCA induced the expression of anti-inflammatory cytokine, Arg-1. In conclusion, TUDCA inhibits inflammatory responses in RAW 264.7 macrophages, BV2 microglial cells, and BMMs. TUDCA can be a potential alternative drug for SCI.

## Introduction

Spinal cord injury (SCI) is a type of severe trauma with poor reversibility and high disability rates^1^. According to the statistics of National Spinal Cord Injury Statistical Center (NSCISC), SCI affects approximately 54 people per million out of the population in the U.S.A. or approximately 17,000 new SCI cases each year^2^. SCI also results in primary and secondary damage, in which inflammation is one of the main causes at the secondary damage^3^.

The inflammatory response is a physiological process against detrimental stimuli such as lipopolysaccharide (LPS) in pathogens which arise due to physical damage^4^. Macrophages and microglia play a critical role in the inflammatory responses through the production of various cytokines^5,6^. Specifically, RAW 264.7 macrophages and BV2 microglial cells are well known cells for inflammatory model in *in vitro*^7–9^. Bone marrow-derived macrophages (BMMs) are primary macrophage cells, derived from rat bone marrow in the presence of growth factors. Activated macrophages and microglia respond to pathogen invasions by producing inflammatory mediators such as nitric oxide (NO)^10–12^ and pro-inflammatory cytokines such as tumor necrosis factor-α (TNF-α), interleukin 1-β (IL-1β), cyclooxygenase-2 (COX-2), and inducible nitric oxide synthase (iNOS)^5,13,14^. Despite the many studies of the inhibition of excessive inflammatory responses in SCI^15–17^, the anti-inflammatory medications nonetheless can have serious side effects^18,19^.

Bear bile has been a component of Asian medicine for thousands of years. It has been used to treat several diseases, ranging from a sore throat to hemorrhoids^20^. Among hydrophilic bile acids, Tauroursodeoxycholic acid (TUDCA) is the major component in the bile acids of the bear^21^. TUDCA is a taurine conjugated form of ursodeoxycholic acid (UDCA), which reduces liver damage in the event of cholestasis^22^. TUDCA is a cytoprotective bile acid having chaperone-like properties, and it constitutes approximately 0.13% of the bile acid pool in human serum^23^. Previous studies have reported that TUDCA reduces endoplasmic reticulum (ER) stress and promotes blood vessel repair^24,25^. Other researchers have investigated the anti-inflammatory effects of TUDCA. However, the studies were only based on microglia cells, not macrophages and BMMs^26,27^. In particular, researchers have not investigated whether TUDCA shows an anti-inflammatory effect in cases involving SCI. Therefore, in this study, we aim to evaluate the anti-inflammatory effects of TUDCA using LPS-stimulated RAW 264.7 macrophages, BV2 microglial cells, and BMMs, and a rat SCI model.

## Results

### Toxicity of TUDCA

**Figure 1:**
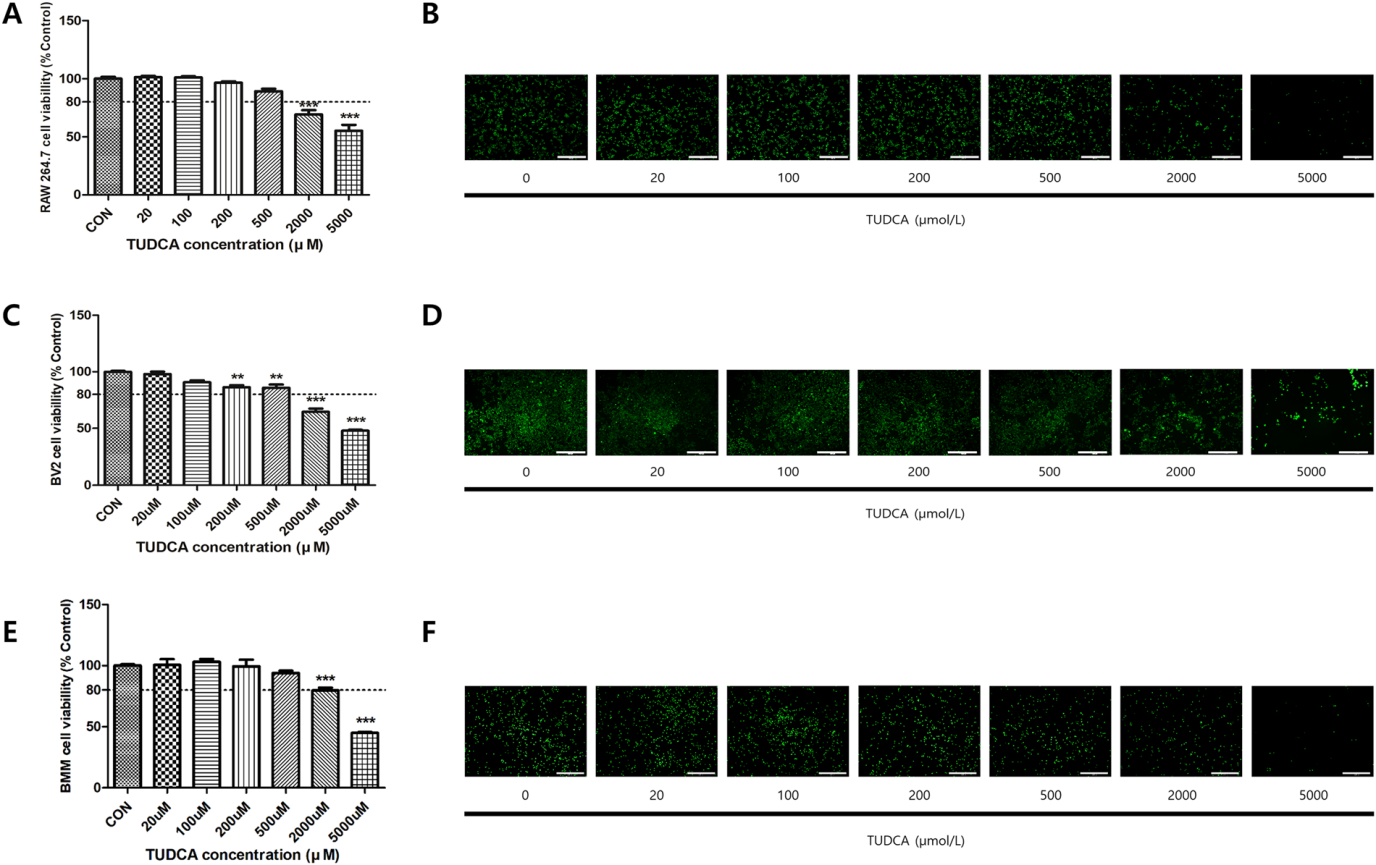
Effect on the cell viability of RAW 264.7 macrophages, BV2 microglial cells, and BMMs. Cell viability was measured using a CCK-8 assay and a live/dead staining kit. (**A**,**B**) The macrophages were treated with each concentration of TUDCA (0, 20, 100, 200, 500, 2000, and 5000 μM) for 24 h. (**C**,**D**) The BV2 microglial cells were treated with each concentration of TUDCA (0, 20, 100, 200, 500, 2000, and 5000 μM) for 24 h. (**E**,**F**) The BMMs were treated with each concentration of TUDCA (0, 20, 100, 200, 500, 2000, and 5000 μM) for 24 h. Results are the mean ± SD of triplicate experiments: **p < 0.01, ***p < 0.001, significant difference as compared to the control group and to each other by one-way ANOVA followed by Tukey’s post hoc analysis.

Cell viabilities (Fig. 1A,C,E) above 80% were noted in the 20, 100, 200, and 500 μM groups for 24 h in RAW 264.7 macrophages (101.28% ± 2.00, 101.01% ± 1.82, 96.56% ± 1.89, and 89.13% ± 3.77, respectively), BV2 microglial cells (97.90% ± 3.77, 90.73% ± 2.91, 86.25% ± 3.20, 85.78% ± 5.09, respectively), and BMMs (100.55% ± 8.25, 103.01% ± 4.30, 99.44% ± 9.21, and 93.90% ± 3.24, respectively). However, the cell viability levels for the 2000 μM and 5000 μM groups were less than 80% (RAW 264.7; 69.17% ± 6.60 and 55.24% ± 8.84, BV2 cells; 64.62% ± 4.88 and 47.93% ± 1.30, BMMs; 79.74% ± 3.74 and 44.91% ± 1.28). Furthermore, the live/dead staining assay results for 24 h in the RAW 264.7 macrophage, microglial cells and BMMs (Fig. 1B,D,F) showed a trend similar to that in the results shown in Fig. 1A,C and E.

### Suppression of NO production by TUDCA

**Figure 2:**
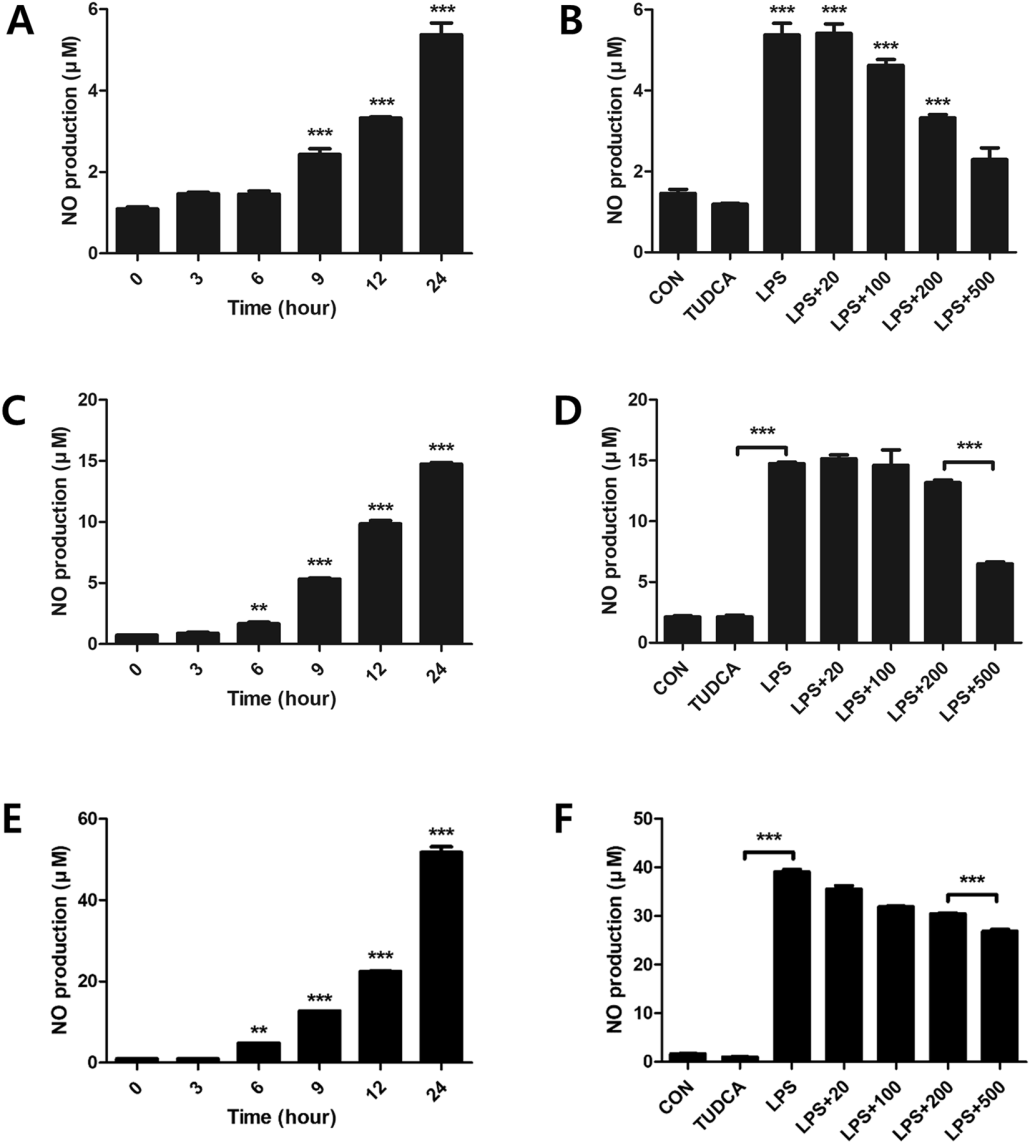
Nitric oxide (NO) in RAW 264.7 macrophages, BV2 microglial cells, and BMMs treated with TUDCA or lipopolysaccharide (LPS) or LPS containing TUDCA. Effect of TUDCA on LPS-induced NO production in Raw 264.7 macrophage, BV2 microglial cells, and BMMs. (**A**,**C**,**E**) The macrophages, microglial cells, and BMMs were treated with LPS for 0, 3, 6, 9, 12, and 24 h. (**B**,**D**,**F**) The macrophages, microglial cells, and BMMs in the LPS alone group were treated with 1 µg/mL of LPS for 24 h. The macrophages in the LPS containing TUDCA group were pre-treated with 20, 100, 200, and 500 µM of TUDCA for 1 h, after which they were treated with LPS (1 µg/mL) containing equal concentrations of TUDCA at the same time. The TUDCA group was treated with 500 µM of TUDCA for 24 h. The collected supernatants were reacted with the Griess reagent, and the absorbance level was measured at 548 nm. Results are the mean ± SD of triplicate experiments: ***p < 0.001, significant difference as compared to the control group by one-way ANOVA followed by Tukey’s post hoc analysis.

With the LPS treatment, NO increased steadily for 24 h, with a maximum index at 24 h (5.37 ± 0.50, 14.73 ± 0.23, 51.76 ± 2.42, respectively, Fig. 2A,C,E). NO in the LPS + TUDCA groups were decreased compared to that in the LPS group in the order of 100 μM (4.62 μM ± 0.25), 200 μM (3.32 μM ± 0.13), and 500 μM (2.29 μM ± 0.49) at 24 h in RAW 264.7 macrophages (Fig. 2B). NO in the LPS + TUDCA groups were decreased compared to that in the LPS group in the order of 100 μM (14.95 μM ± 2.51), 200 μM (13.16 μM ± 0.37), and 500 μM (6.49 μM ± 0.28) at 24 h in BV2 microglial cells (Fig. 2D). NO in the LPS + TUDCA groups were decreased compared to that in the LPS group in the order of 100 μM (31.90 μM ± 0.26), 200 μM (30.45 μM ± 0.29), and 500 μM (26.83 μM ± 0.77) at 24 h in BMMs (Fig. 2F).

### Reduced mRNA expression of inflammatory cytokines by TUDCA

**Figure 3:**
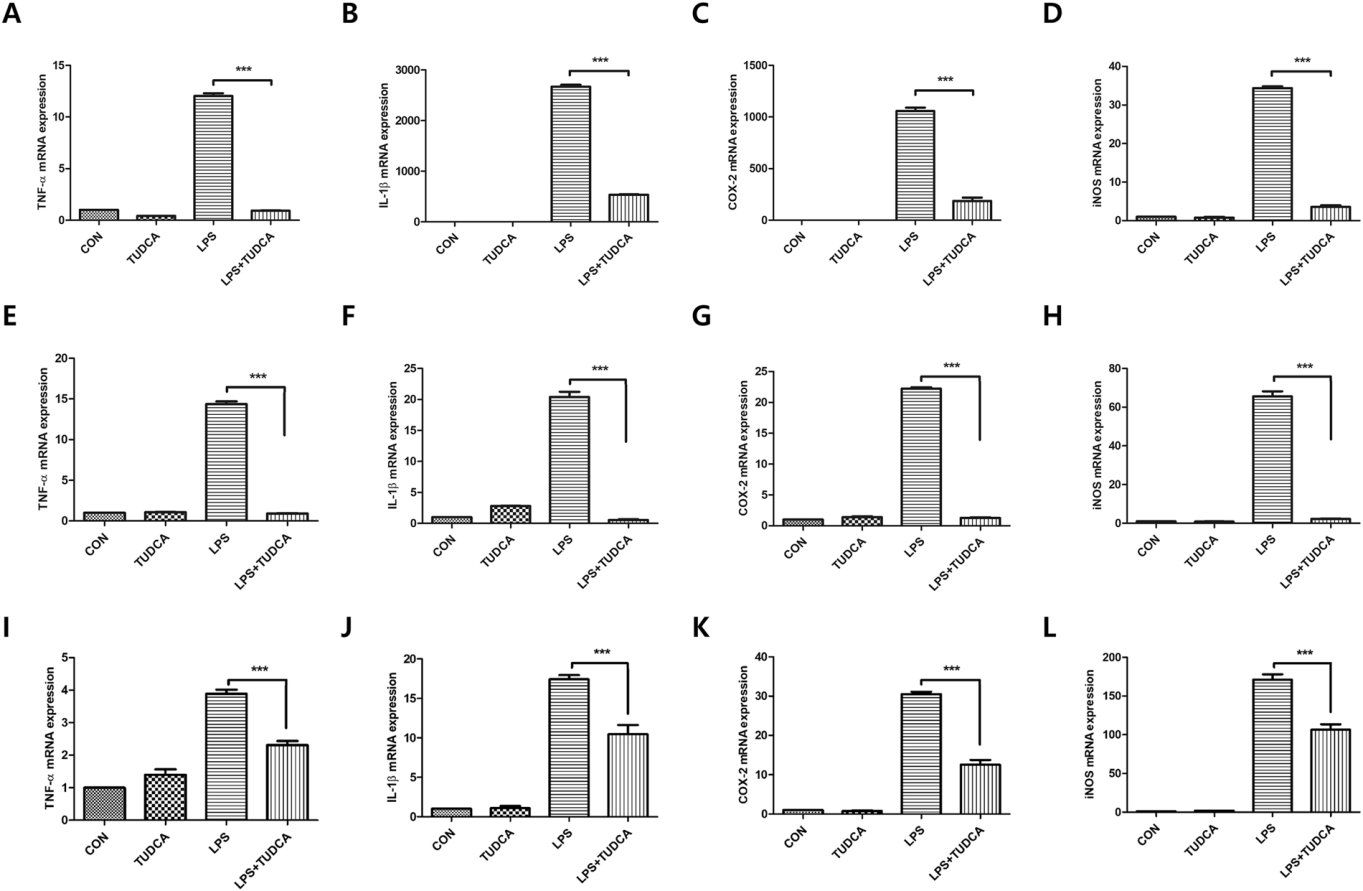
The mRNA expression in RAW 264.7 macrophages, BV2 microglial cells, and BMMs treated with TUDCA (500 µM) or LPS (1 µg/mL) or LPS containing TUDCA. The macrophages, BV2 microglial cells, and BMMs in the TUDCA group and the LPS + TUDCA group were pretreated with 500 µM TUDCA for 1 h before the zero time point. Then, LPS + TUDCA group was treated with LPS (1 µg/mL) containing 500 µM of TUDCA for 24 h. The TUDCA group was treated with 500 µM of TUDCA. The macrophages, BV2 microglial cells, and BMMs in the LPS group were treated with 1 µg/mL of LPS for 24 h. Cell pellets were subjected to a qRT-PCR analysis to detect the expression levels of (**A**,**E**,**I**) TNF-α, (**B**,**F**,**J**) IL-1β (**C**,**G**,**K**) COX-2, and (**D**,**H**,**L**) iNOS mRNA. The levels of each mRNA expression were referenced to the expression of GAPDH mRNA. The fold ratio of the control group was set at 1-fold and the relative fold change was calculated. Results are the mean ± SD of triplicate experiments ***p < 0.001, significant difference as compared to each other by one-way ANOVA followed by Tukey’s post hoc analysis.

Figure 3A–D indicates that 500 μM of TUDCA decreased inflammatory cytokines in LPS-stimulated RAW 264.7 macrophages significantly in the mRNA levels. The expressions of TNF-α, IL-1β, COX-2 and iNOS were significantly reduced in the LPS + TUDCA group as compared to the reduction in the LPS group (TNF- α; 12.03 ± 0.45 vs 0.91 ± 0.08, IL-1β; 2668.32 ± 65.62 vs 533.70 ± 14.10, COX-2; 1058.25 ± 55.06 vs 186.53 ± 56.59, iNOS; 34.38 ± 0.77 vs 3.55 ± 0.67, respectively, Fig. 3A–D). 500 μM of TUDCA also significantly decreased pro-inflammatory cytokines in LPS-stimulated BV2 microglial cells in the mRNA levels. The expressions of TNF-α, IL-1β, COX-2 and iNOS were significantly reduced in the LPS + TUDCA group as compared to the reduction in the LPS group (TNF- α; 14.36 ± 0.58 vs 0.90 ± 0.07, IL-1β; 20.38 ± 1.48 vs 0.56 ± 0.18, COX-2; 22.21 ± 0.38 vs 1.26 ± 0.18, iNOS; 65.59 ± 4.46 vs 2.24 ± 0.36, respectively, Fig. 3E–H). 500 μM of TUDCA also significantly decreased pro-inflammatory cytokines in LPS-stimulated BMMs in the mRNA levels. The expressions of TNF-α, IL-1β, COX-2 and iNOS were significantly reduced in the LPS + TUDCA group as compared to those in the LPS group (TNF- α; 3.88 ± 0.28 vs 2.30 ± 0.21, IL-1β; 17.40 ± 0.92 vs 10.44 ± 2.02, COX-2; 30.49 ± 1.05 vs 12.56 ± 2.11, iNOS; 171.15 ± 12.14 vs 106.39 ± 12.45, respectively, Fig. 3I–L).

### Reduced protein expression of inflammatory cytokines by TUDCA

**Figure 4:**
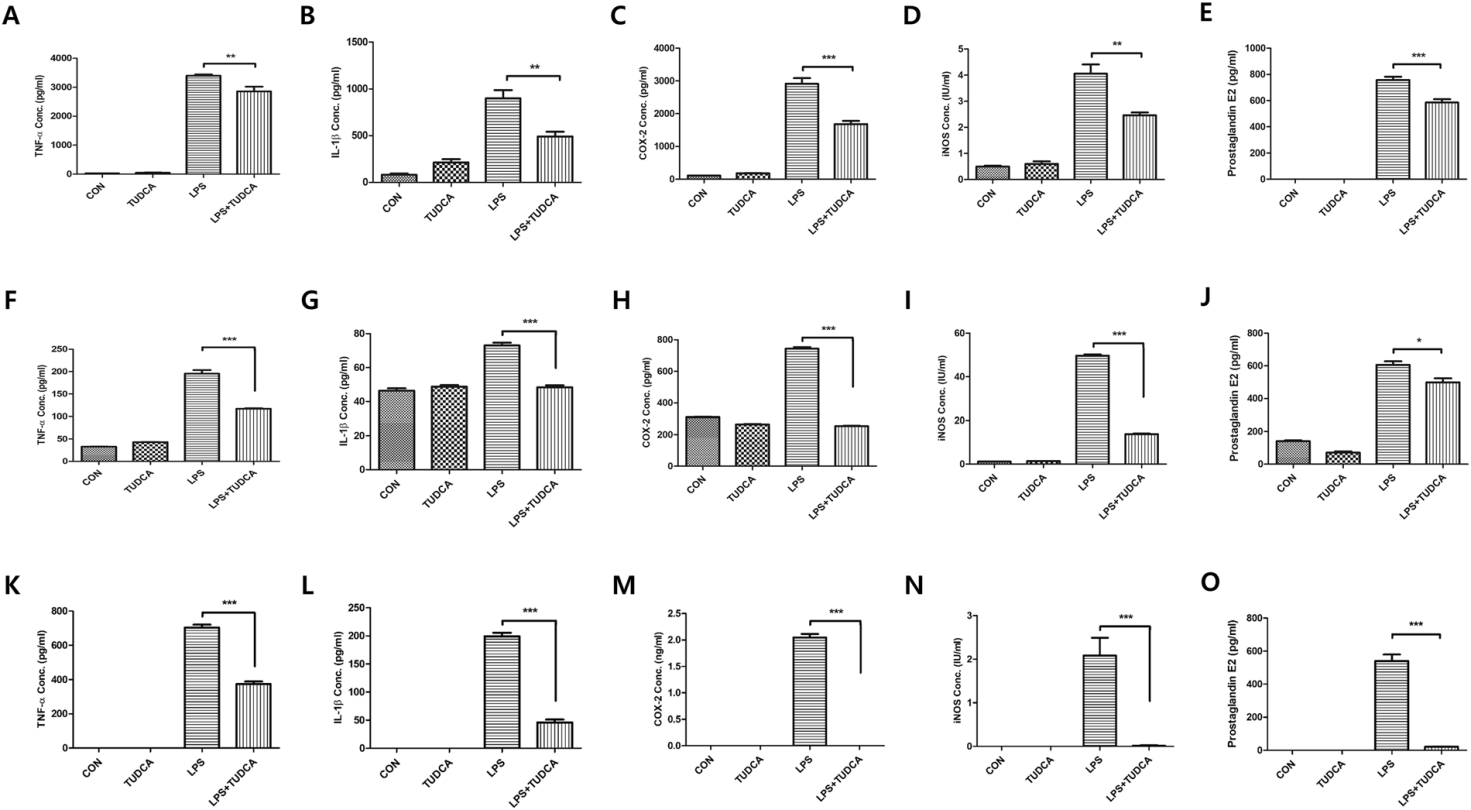
The protein concentration in RAW 264.7 macrophages, BV2 microglial cells, and BMMs treated with TUDCA (500 µM) or LPS (1 µg/mL) or LPS containing TUDCA. The macrophages, BV2 microglial cells, and BMMs in the TUDCA group and in the LPS + TUDCA group were pretreated with 500 µM of TUDCA for 1 h before the zero time point. The LPS + TUDCA group was then treated with LPS (1 µg/mL) containing 500 µM of TUDCA for 24 h. The TUDCA group was treated with 500 µM TUDCA. The macrophages, BV2 microglial cells, and BMMs in the LPS group were treated with 1 µg/mL of LPS for 24 h. The collected supernatants were subjected to the plate of the ELISA kit to detect the proteins of (**A**,**F**,**K**) TNF-α, (**B**,**G**,**L**) IL-1β, (**C**,**H**,**M**) COX-2 (**D**,**I**,**N**) iNOS, and (**E**,**J**,**O**) PGE_2_. Results are the mean ± SD of triplicate experiments: *p < 0.05, **p < 0.01, and ***p < 0.001 significant difference as compared to each other by one-way ANOVA followed by Tukey’s post hoc analysis.

TUDCA at 500 μM TUDCA significantly decreased inflammatory cytokines in LPS-stimulated RAW 264.7 macrophages, BV2 microglial cells, and BMMs in protein levels at 24 h (Fig. 4). In RAW 264.7 macrophages, the protein expressions of TNF-α, IL-1β, COX-2 and iNOS were significantly reduced in the LPS + TUDCA group compared to the LPS group (TNF-α; 3402.35 pg/ml ± 65.67 vs 2860.52 pg/ml ± 281.33, IL-1β; 893.32 pg/ml ± 150.48 vs 483.46 pg/ml ± 88.90, COX-2; 2910.51 pg/ml ± 301.66 vs 1682.34 pg/ml ± 168.25, iNOS; 4.05 IU/ml ± 0.61 vs 2.45 IU/ml ± 0.18, respectively, Fig. 4A–D). In BV2 microglial cells, the protein expressions of TNF-α, IL-1β, COX-2 and iNOS were significantly reduced in the LPS + TUDCA group compared to the LPS group (TNF-α; 195.27 pg/ml ± 13.95 vs 116.76 pg/ml ± 2.34, IL-1β; 73.05 pg/ml ± 2.76 vs 48.38 pg/ml ± 2.02, COX-2; 743.62 pg/ml ± 17.25 vs 254.35 pg/ml ± 4.16, iNOS; 50.24 IU/ml ± 0.41 vs 13.85 IU/ml ± 0.53, respectively, Fig. 4F–I). In BMMs, the protein expressions of TNF-α, IL-1β, COX-2 and iNOS were significantly reduced in the LPS + TUDCA group compared to those in the LPS group (TNF-α; 703.99 pg/ml ± 28.47 vs 374.51 pg/ml ± 24.31, IL-1β; 199.19 pg/ml ± 10.83 vs 45.91 pg/ml ± 8.78, COX-2; 2.04 ng/ml ± 0.11 vs 0 ng/ml, iNOS; 2.08 IU/ml ± 0.70 vs 0.02 IU/ml ± 0.03, respectively, Fig. 4K–N). In addition, PGE_2_ in the end product of COX-2 was reduced in the LPS + TUDCA group more than it was in the LPS group (RAW 264.7 cells; 756.68 pg/ml ± 44.29 vs 585.72 pg/ml ± 43.76, BV2 cells; 605.46 pg/ml ± 38.70 vs 498.91 pg/ml ± 42.86, BMMs; 540.10 pg/ml ± 68.06 vs 21.38 pg/ml ± 2.71, Fig. 4E,J and O).

### Histological effect of TUDCA and Behavior test

**Figure 5:**
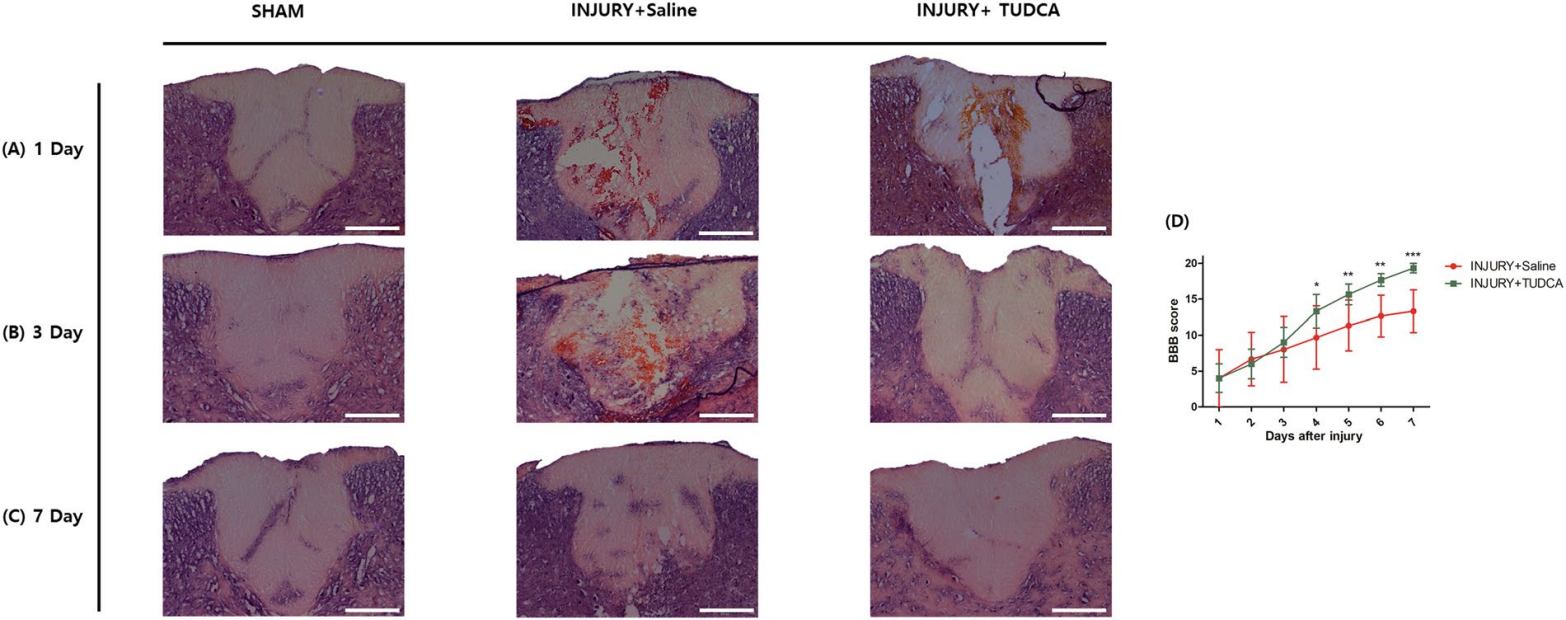
Histopathological effect of TUDCA by H&E staining and behavior tests in injury and TUDCA group. Lesion epicenter histopathology (H&E staining): (**A**) Spinal cord tissue on 1 day after spinal cord injury. (**B**) Spinal cord tissue on 3 day after the injury shows more remaining neurons and low inflammation in the TUDCA group. (**C**) Spinal cord tissues on 7 day after the injury are similar in the TUDCA and sham groups (Scale bar = 200 µm). (**D**) The Basso-Beattie-Bresnahan (BBB) scores for the hindlimb function were evaluated for 7 days after the injury. The TUDCA group received intraperitoneal injections of 200 mg/kg of TUDCA at one minute and 24 hours after establishing the model. The injury group received intraperitoneal injections of the same dose of saline at the same time points. Results are the mean ± SD of triplicate experiments: *p < 0.05, **p < 0.01, and ***p < 0.001, significant difference as compared to the 1 day for each group by unpaired Student’s t-test.

On 1 day after SCI, the spinal cord tissues showed large areas of hemorrhage, edema, gray matter structural damage, and vacuolar degeneration in the injury group. The TUDCA group did not show distinct histopathology differences (Fig. 5A). On 3 day after SCI, however, the lesion epicenter showed obvious structural damage in the injury group. In contrast, the lesion epicenter of the TUDCA group showed much milder damage (Fig. 5B). On 7 day after SCI, the lesion epicenter of the injury group still showed cavities. On the other hand, the lesion epicenter of the TUDCA group was nearly normal in appearance (Fig. 5C).

The Basso-Beattie-Bresnahan (BBB) scores for the hindlimb motor function in the injury group improved from 4.00 ± 6.92 at 1 day to 13.33 ± 5.13 at 7 day. The scores in the TUDCA group demonstrated a significant improvement from 4.00 ± 3.46 at 1 day to 19.33 ± 1.15 at 7 day after SCI (***p < 0.001). The differences of the scores in the TUDCA group were significant after 4 day (Fig. 5D).

**Figure 6:**
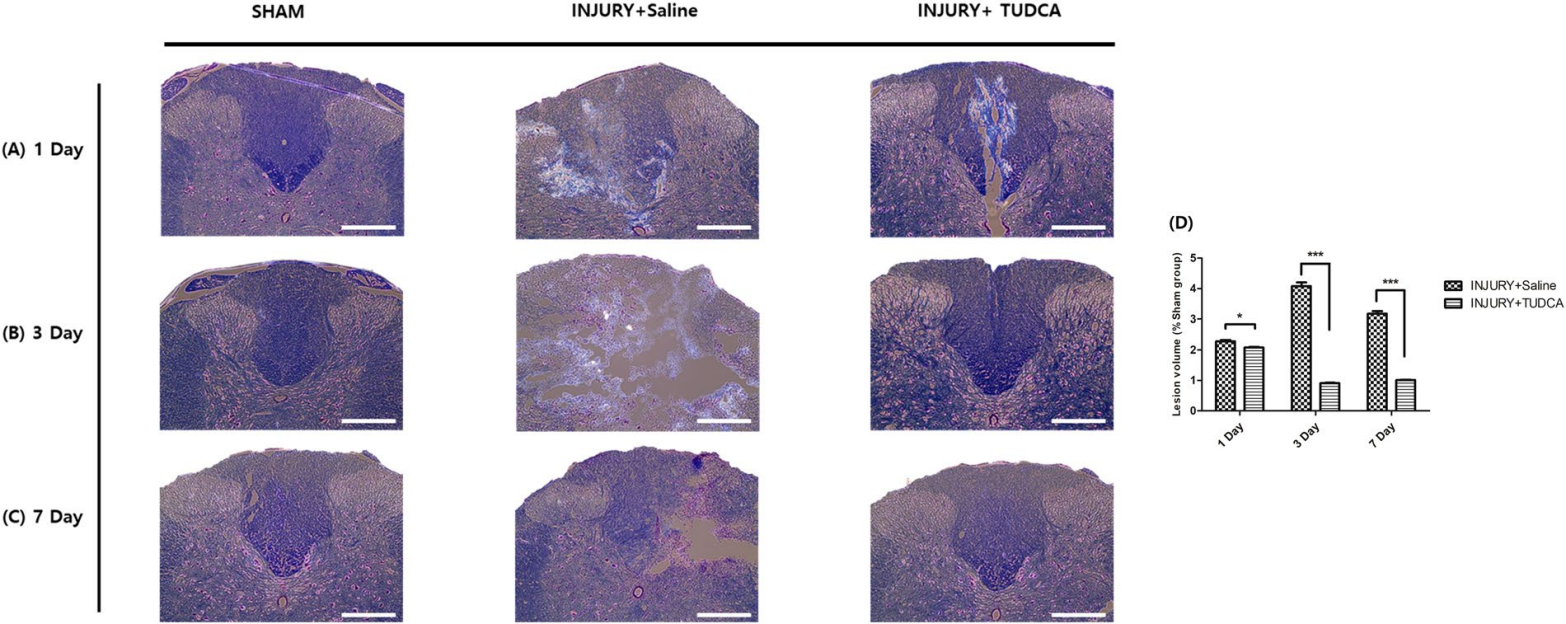
Histopathological effect of TUDCA by Luxol Fast Blue/cresyl violet-staining. Lesion epicenter histopathology (luxol fast blue/cresyl violet-staining staining): (**A**) Spinal cord tissue on 1 day after spinal cord injury. (**B**) Spinal cord tissue on 3 day after the injury shows more remaining myelin fiber and observes nerve cells in the TUDCA group. (**C**) Spinal cord tissues on 7 day after the injury are similar in the TUDCA and sham groups (Scale bar = 200 µm). Results are the mean ± SD of triplicate experiments: *p < 0.05, **p < 0.01, and ***p < 0.001 by one-way ANOVA followed by Tukey’s post hoc analysis.

Lesion volume was then assessed by luxol fast blue/cresyl violet-stained spinal cord sections serially in the axial plane. In injury group, the lesions often contained vacuoles (Fig. 6), which exhibited complete loss of myelinated fibers, neurons, oligodendrocytes, and astrocytes. However, the lesion volume was significantly decreased in the injury + TUDCA group on 3 day and 7 day (0.90% ± 0.03, 1.01% ± 0.02, respectively) compared to that of the injury group (4.08% ± 0.20, 3.17% ± 0.02, respectively, Fig. 6D).

### Fluorescence expression effect of TUDCA

**Figure 7:**
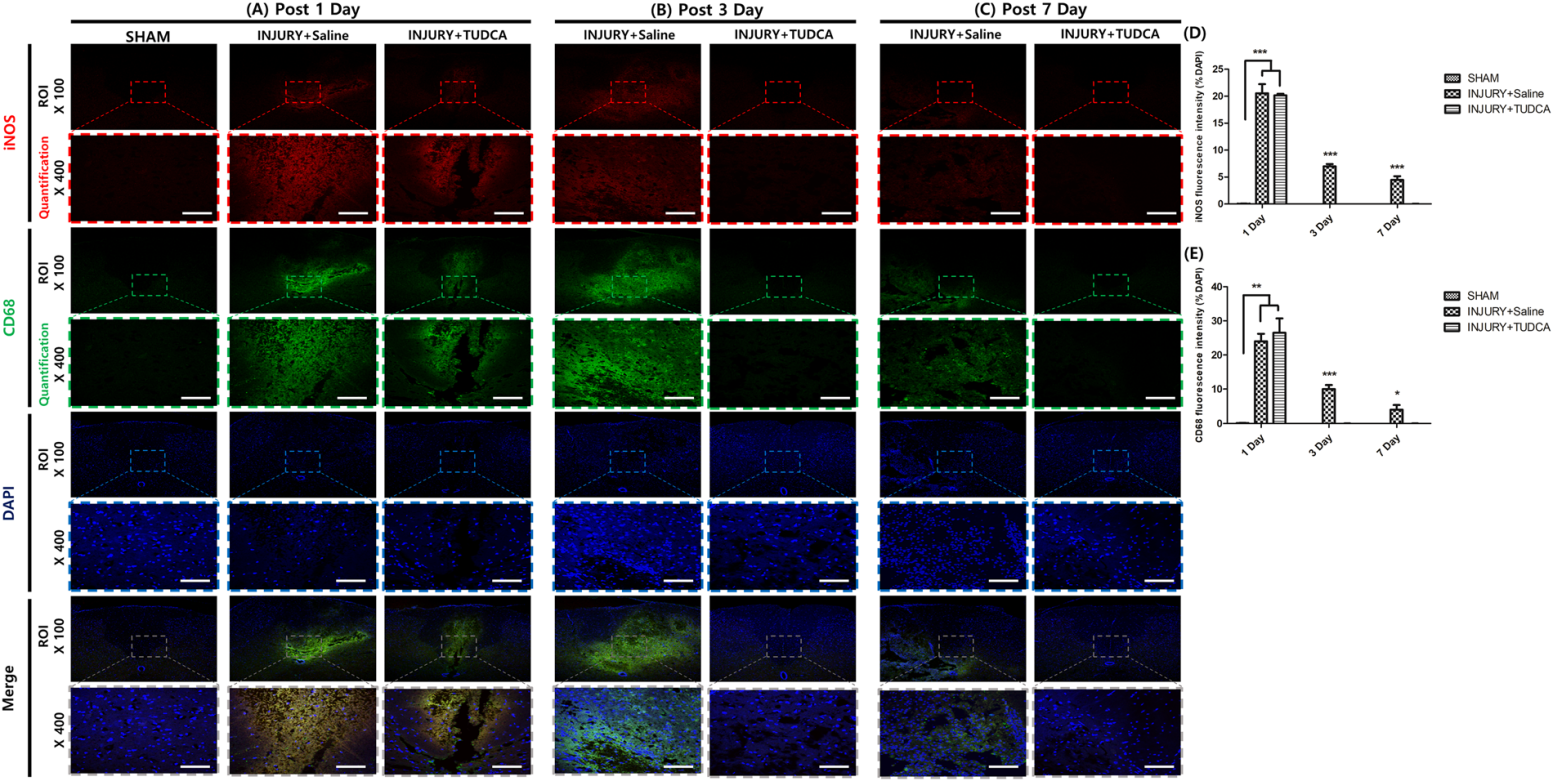
Immunofluorescent staining for CD68 and iNOS. Lesion epicenter immunofluorescent staining: (**A**) On 1 day after SCI, the expressions of CD68 and iNOS were not significantly different. (**B**) On 3 day after SCI, the expressions of CD68 and iNOS were significantly suppressed by TUDCA. (**C**) On 7 days after SCI, the expressions of CD68 and iNOS were significantly suppressed by TUDCA (Scale bar = 80 µm). (**D**) Quantitative analysis of the fluorescence intensity for iNOS. (**E**) Analysis of the quantitative fluorescence intensity for CD68. Results are the mean ± SD of triplicate experiments: *p < 0.05, **p < 0.01, and ***p < 0.001 significant difference as compared to the control group for each time by one-way ANOVA followed by Tukey’s post hoc analysis.

**Figure 8:**
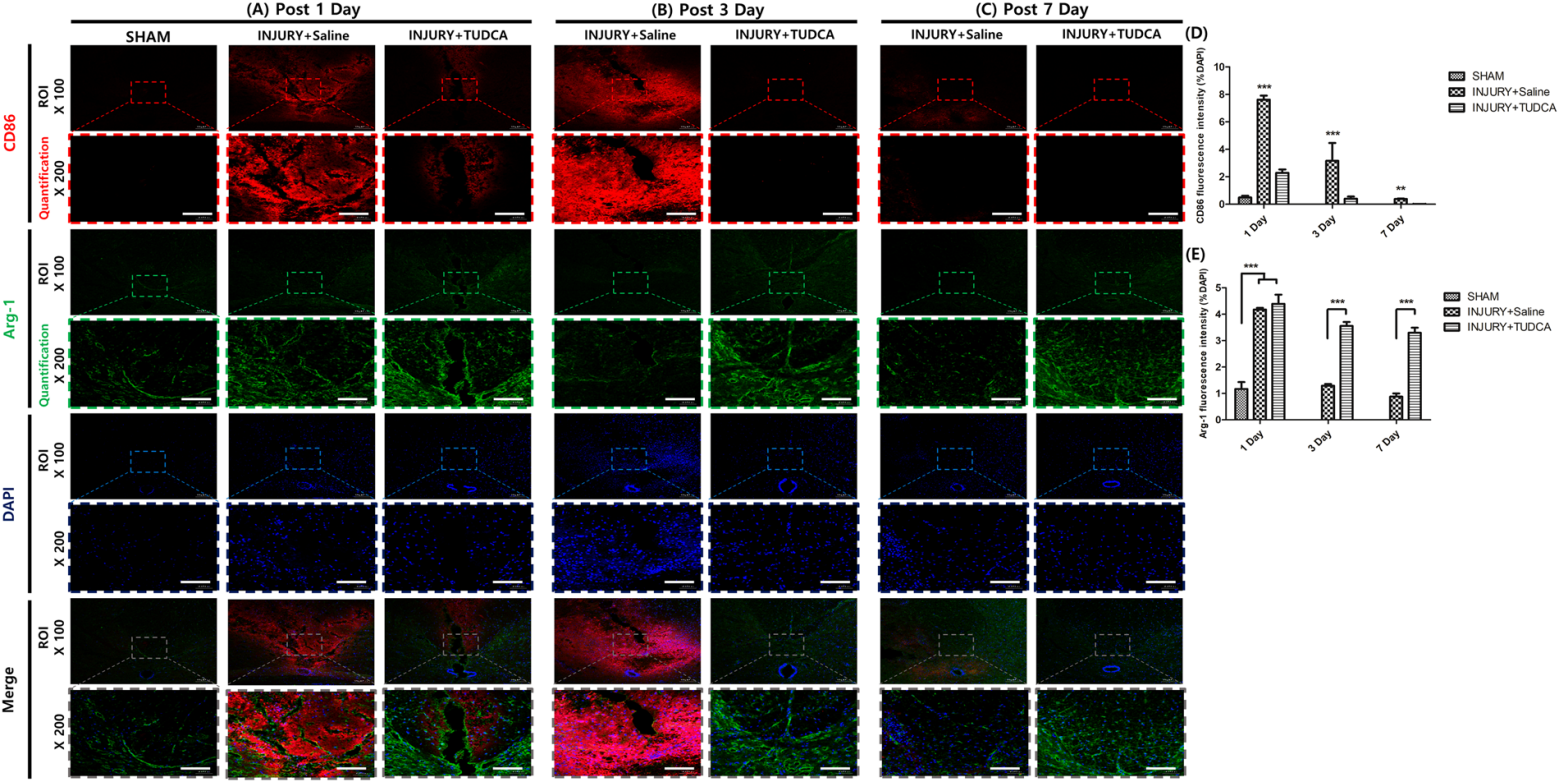
Immunofluorescent staining for CD86 and Arg-1. Lesion epicenter immunofluorescent staining: (**A**) On 1 day after SCI, the expressions of CD86 was significantly suppressed by TUDCA. However, the expressions of Arg-1 were not significantly different. (**B**) On 3 day after SCI, the expressions of CD86 were significantly suppressed by TUDCA and the expressions of Arg-1 were significantly induced by TUDCA. (**C**) On 7 days after SCI, the expressions of CD86 were significantly suppressed by TUDCA and the expressions of Arg-1 were significantly induced by TUDCA. (Scale bar = 80 µm). (**D**) Quantitative analysis of the fluorescence intensity for CD86. (**E**) Analysis of the quantitative fluorescence intensity for Arg-1. Results are the mean ± SD of triplicate experiments: **p < 0.01, and ***p < 0.001 significant difference as compared to the control group for each time by one-way ANOVA followed by Tukey’s post hoc analysis.

In the sham group, CD68 positive cells and iNOS were not detected at any time. On 1 day after SCI, the TUDCA group did not significantly differ from the injury group regarding CD68, iNOS, and Arg-1 expression levels (Figs 7A,D,E and 8E) However, the fluorescence intensities of CD86 were significantly decreased in the TUDCA group compared to those in the injury group (Fig. 8A,D). On 3 day after SCI, the fluorescence intensities of CD68, iNOS and CD86 were significantly decreased in the TUDCA group compared to those in the injury group. On the other hand, the fluorescence intensities of Arg-1 were significantly increased in the TUDCA group compared to those in the injury group. On 7 day after SCI, the fluorescence intensities of CD68, iNOS and CD86 were also significantly decreased in the TUDCA group relative to those in the injury group (CD68: ***p < 0.001, iNOS: *p < 0.05, CD86: ***p < 0.001, Figs 7B–E and 8B–D). The fluorescence intensities of Arg-1 were also significantly increased in the TUDCA group compared to those in the injury group (Arg-1: ***p < 0. 001, Fig. 8B,C,E).

## Discussion

In this study, the cell viability, NO, qRT-PCR, and ELISA results demonstrated that the inflammatory responses in LPS-stimulated RAW 264.7 macrophages, BV2 microglia cells, and BMMs were effectively inhibited in the 500 μM TUDCA group without cytotoxicity. In addition, through H&E staining, luxol fast blue/cresyl violet-staining and immunofluorescent staining, we observed the anti-inflammatory and potential therapeutic effect of TUDCA after SCI in rats.

To determine the cytotoxicity concentration of TUDCA, RAW 264.7 macrophages, BV2 microglial cells, and BMMs were treated with TUDCA at each concentration ranging from 0 μM to 5000 μM for 24 h and CCK-8 assays were conducted for quantitative measurements. Cell viability exceeding 80% was regarded as evidence of non-toxic density levels^28^, and we found that TUDCA had no cytotoxicity up to 500 μM (Fig. 1).

The principal inflammatory mediator, NO, was produced in LPS-stimulated RAW 264.7 macrophage cells, BV2 microglial cells, and BMMs. In particular, NO production increased consistently for 24 h (Fig. 2A,C and E). Figure 2B,D and F show that TUDCA effectively inhibited NO production at 500 µM in LPS-stimulated RAW 264.7 macrophage, BV2 microglia, and BMMs. Therefore, we decided to use 500 μM of TUDCA in the subsequent experiments.

Macrophages and microglia have several major functions such as antigen presentation, phagocytosis, and immunomodulation through the production of various cytokines during the inflammatory process^4,6^. Natalia *et al*. reported that TUDCA suppressed the expression of NO and iNOS^26,27^. However, they used microglial cells and did not find other cytokines apart from NO and iNOS. We observed that TUDCA suppressed the pro-inflammatory cytokines of TNF-α IL-1β, COX-2, iNOS and PGE_2_ (Figs 3 and 4) in LPS-stimulated RAW 264.7 macrophages and BV2 microglial cells.

After SCI, the spinal cord can show large areas of hemorrhage, edema^29^. Our histopathological results also demonstrated the types of changes (Figs 5 and 6). On day 1 after SCI, H&E staining and luxol fast blue/cresyl violet-staining of the injury and TUDCA groups did not show significant differences in our study. However, H&E staining and luxol fast blue/cresyl violet-staining of the TUDCA group showed an obvious improvement in the structural damage after 3 days. These staining results were in accordance with the findings of a previous study^30,31^.

Macrophages and microglia polarizations are sometimes categorized into classical (M1) and alternative (M2) activation^32^. M1 macrophages and microglial cells express pro-inflammatory molecules of TNF-a, IL-1β, COX-2, iNOS and NO. They also express cell surface markers, CD86 and CD68. On the other hand, M2 macrophages and microglial cells express anti-inflammatory molecule of Arg-1. The Arg-1 shows neuroprotective effects in SCI rats^33^.

An investigation with immunofluorescent staining showed that CD68, iNOS and CD86 were significantly reduced starting at 3 days in our study. In a previous study, apoptosis was decreased even on 1 day after SCI^26^. Anti-inflammation markers may be improved later than apoptosis marker, but further comparative studies are warranted to elucidate the time of effect after the treatment.

Although we need to compare steroidal anti-inflammatory drug with TUDCA, to the best of our knowledge, our study is the first to show that TUDCA inhibits the inflammatory response in RAW 264.7 macrophages, BMMs, and that TUDCA decreases inflammation in a SCI model.

## Conclusion

TUDCA inhibits the LPS-stimulated inflammatory response in RAW 264.7 macrophages, BV2 microglia cells, and BMMs. TUDCA suppresses expression of inflammatory cytokines in SCI rat model. The results here suggest that TUDCA can serve as a useful anti-inflammatory drug and that it is potential alternative drug for SCI.

## Materials and Methods

### Main Reagents

TUDCA was obtained from TCI (Tokyo Chemical Industry Co., Tokyo, Japan) and solubilized in Dulbecco’s modified Eagle’s medium (DMEM, GIBCO, Grand Island, USA) containing 10% fetal bovine serum (FBS, GIBCO) and 1% penicillin-streptomycin (PS, GIBCO) for each experimental concentration. LPS was purchased from Sigma Aldrich (Sigma, St. Louis, USA) and melted with distilled water (100 μg/ml), after which it was diluted with DMEM (1 μg/ml) to induce an inflammatory response in RAW 264.7 macrophage cells.

### Experimental group

We compared the differences among 4 groups: a no treatment group in RAW 264.7 macrophages (control group), a TUDCA only treatment group (TUDCA group), a LPS only treatment group (LPS group), and a LPS containing TUDCA treatment group (LPS + TUDCA group).

### Cell culture

RAW 264.7 macrophages were obtained from the Korean Cell Lines Bank (KCLB, Seoul, Korea). BV2 microglial cells were obtained from the ATCC (Manassas, VA). RAW 264.7 macrophages and BV2 microglial cells were cultured in DMEM containing 10% FBS and 1% Penicillin at 37 °C in a 5% CO_2_ atmosphere. Media was changed once a day in all experiments.

### Isolation of Bone marrow-derived macrophages and primary culture

The isolation protocol from rat bone marrow by Ahmed *et al*.^34,35^ was used. Shortly, rats were euthanized by CO_2_ asphyxiation, and femur and tibia were collected aseptically. Bones were cut in half and placed in a 1.5 ml reaction tube. Bone marrow was collected by centrifugation (5 min, 5000 × g). Cells were separated with a 40 μm cell strainer, and erythrocytes were lysed via hypotonic shock in sterile distilled water. To analyse adhesion and proliferation, we used an established protocol to enrich BMMs from whole bone marrow by their ability of attachment to untreated plastic^5^. Separation via adhesion to plastic provides easy and reliable access to the macrophage proportion of bone marrow cells, and the obtained population is further considered to be BMMs. After centrifugation (5 min, 245 × g), remaining bone marrow cells were suspended in 20 ml of macrophage medium consisting of alpha-minimum essential medium (α-MEM) with 10% FBS, 2% glutamate and 1% penicillin and 20 ng/ml recombinant rat macrophage colony-stimulating factor (M-CSF) (#315-02; Peprotech, NY, USA). Five milliliters of the suspension was placed in a 100 × 20 mm petri dish (#430591; Corning, NJ, USA) and cultivated at 37 °C and 5% CO_2_. After 2 days of preculture. Afterwards, plates were washed with Dulbecco’s phosphate-buffered saline (DPBS, Invitrogen), and all non-adherent cells in the supernatant were removed. Attached cells, regarded as macrophages, were detached by using 0.02% ethylene diamine tetraacetic acid (EDTA) in DPBS. Subsequently, 5 min incubation period on ice was followed by 1 min at −20 °C and a cell scraper. After centrifugation and resuspension in α-MEM, the cell number was determined with a hemocytometer and macrophages were seeded at a density of 1.5 × 10^4^ cells/cm^2^ for indicated experiments.

### Cell viability

Cell viability at each TUDCA density (0, 20, 100, 200, 500, 2000, 5000 μM) was evaluated using a cell counting kit (CCK-8, Dojindo Molecular Technologies Inc., Japan) and a live/dead staining kit (Invitrogen, Carlsbad, USA). After seeding the cells on a 12-well culture plate (Falcon, 1 × 10^5^ cells/well, n = 3 per group), the adhering and proliferating cells were measured. At 24 h, cells were washed with DPBS After the addition of fresh media containing CCK-8 (500 μL of 0.1 mL/ml), the cells were incubated for 2 h. After incubation, the intensity was measured by a microplate reader (Bio-Rad, Hercules, USA) at a wavelength of 450 nm. The absorbance of the control group was fixed at 100% and the absorbance levels of other groups were calculated relative to that level. Under identical conditions, cells were stained with calcein-AM/ethidium homodimer-1 (EthD-1) from live/ dead staining kit. After reacting for 15 min, the cells in all groups were observed under 40× magnification using an inverted fluorescence microscope (Olympus IX71, Tokyo, Japan).

### Measurement of NO production

In the LPS group (1 × 10^5^ cells/well, n = 3 per group), cells on a 48-well plate (Falcon) were treated by LPS (1 μg/mL) at 0, 3, 6, 9, 12, 24 h. Base on the results, we decided on 24 h as a LPS treatment time point. The LPS + TUDCA group was pretreated with each concentration of TUDCA (0, 20, 100, 200, 500 μM) for 1 h. The LPS + TUDCA group was stimulated with LPS containing equal concentrations of TUDCA. The accumulated NO was detected using a Griess Reagent Kit for Nitrite Determination (Invitrogen). Equal volumes of the supernatant and sulfanilamide were mixed and incubated for 10 min at room temperature, after which a solution of naphthylethylenediamine dihydrochloride was added. The mixture was then incubated for an additional 5 min. Absorbance was measured at 548 nm, using a calibration curve with sodium nitrite as a standard.

### Quantitative Real Time - Polymerase Chain Reaction (qRT-PCR)

Raw 264.7 macrophages, BV2 microglial cells, and BMMs (1 × 10^5^ cells/well) were seeded on a 48-well plate culture plate (Falcon) and proliferated with TUDCA or LPS or LPS containing TUDCA. The cells were pretreated with 500 μM of TUDCA for 1 h before the zero time point. At the predetermined time points, seeded cells of total RNA were extracted using Trizol reagent (Invitrogen) according to the manufacturer’s instructions. Complementary DNA (cDNA) was synthesized from 1 μg of total RNA using a cDNA using synthesis kit (TAKARA, Shiga, Japan). The qRT-PCR step was performed using an ABI Step One Real-time PCR System (Applied Biosystems, Warrington, UK) and a reaction mixture that consisted of SYBR Green 2 × PCR Master Mix, a cDNA template, and forward and reverse primers. The primers of the measured mRNA genes were as follows: mouse TNF-α; 5′ – AGC AAA CCA CCA AGT GGA GGA − 3′ (sense) and 5′ – GCT GGC ACC ACT AGT TGG TTG T - 3′ (antisense), mouse IL-1β; 5′– TTG TTG CTG TGG AGA AGC TGT - 3′ (sense) and 5’ – AAC GTC ACA CAC CAG CAG GTT - 3′ (antisense), mouse COX-2; 5′ – CGG AGG AGA AGT GGG GTT TAG GAT - 3′ (sense) and 5′ – TGG GAG GCA CTT GCG TTG ATG G -3′ (antisense), mouse iNOS; 5′ – GAC CAG ATA AGG GCA AGC AC - 3′ (sense) and 5′ – CTT GTC TTT GAC CCA GTA GC - 3′ (antisense), mouse GAPDH; 5′ – ATG ATT CTA CCC ACG GCA AG - 3′ (sense) and 5′ CTG GAA GAT GGT GAT GGG TT - 3′ (antisense), rat TNF-α; 5′ – CGT CGT AGC AAA CCA CCA AG - 3′ (sense) and 5′ – CAC AGA GCA ATG ACT CCA AA - 3′ (antisense), rat IL-1β; 5′ – CCC TGC AGC TGG AGA GTG TG - 3′ (sense) and 5’ – TGT GCT CTG CTT GAG AGG TG- 3′ (antisense), rat COX-2; 5′ – CGG AGG AGA AGT GGG GTT TA - 3′ (sense) and 5′ – TGG GAG GCA CTT GCG TTG AT - 3′ (antisense), rat iNOS; 5′ – CAG CGC ATA CCA CTT CAG C - 3′ (sense) and 5′ – ACC ATG GAG CAT CCC AAG - 3′ (antisense), rat GAPDH; 5′ – CAG CGC ATA CCA CTT CAG C - 3′ (sense) and 5′ ACC ATG GAG CAT CCC AAG - 3′ (antisense). The PCR protocol consisted of 40 cycles of denaturation at 95 °C for 15 sec, followed by 60 °C for 30 s to allow for extension and amplification of the target sequence. The relative expression levels of TNF-α, IL-1β, COX-2, and iNOS were normalized to that of glyceraldehyde 3-phosphate dehydrogenase (GAPDH) using the 2^−ΔΔCT^ method. The primers were obtained from Bioneer (Daejeon, Korea).

### Cytokine measurement by Enzyme-Linked ImmunoSorbent Assay (ELISA)

The cells (1 × 10^5^ cells/well, n = 3 per group) on a 96-well plate (Falcon) were pretreated with 500 μM of TUDCA for 1 h. In LPS containing TUDCA group, the cells were stimulated with LPS (1 μg/mL) containing 500 μM of TUDCA for 24 h. PGE_2_ (R&D Systems, Minneapolis, USA), COX-2, iNOS (CUSABIO, Hubei, China), TNF-α, and IL-1β (Koma Biotech, Seoul, South Korea) were measured according to the each manufacturer’s directions.

### Animals

All experiments were performed according to the approved protocol by the Institutional Animal Care and Use Committee (IACUC) of CHA University (IACUC160074) and the Guide for the Care and Use of Laboratory Animals (National Institutes of Health, Bethesda, MD, USA). All experimental procedures related to the animals were conducted in accordance with the declaration by Basic Science Research Program through the National Research Foundation of Korea (NRF) (NRF-2016M3A9E8941668). Thirty one female clean-grade Sprague Dawley rats that were approximately nine weeks old which weighted 170-210 g were provided by Orient Bio Inc. (Seongnam-si, Korea). The rats were housed in a temperature controlled (22 ± 2 °C) animal facility with a light:dark cycle of 12:12 h. The twenty seven rats were randomly divided into a sham group (sham surgery group, n = 9), an injury group (saline treatment group, n = 9) and a TUDCA group (TUDCA treatment group, n = 9). The other four rats were used for isolation of BMMs.

### Establishment of the spinal cord injury model

Rats in the sham group were anesthetized via an intraperitoneal injection of a combination of 10 mg/kg of Rompun (Bayer Animal Health Co, Suwon-si, Korea) and 50 mg/kg of Zoletil 50 (Virbac Laboratories, Carros, France). A midline incision was made in the lower back under sterile conditions, and tissues were dissected layer by layer to reveal the T8-T10 vertebra. A T9 total laminectomy was then conducted to expose the dura. The dura of the rats in the injury group and TUDCA group was exposed as described above, and the spinous processes of the T8 and T10 vertebra were fixed by clamps. A stainless steel rod with a diameter of 2.5 mm and a weight of 35 g was compressed for 5 min. After generating the injury, the weight was quickly removed, and the tissue was sutured. The rats were kept warm and housed separately, with free access to food. On each day at 8 am and 8 pm, bladder massage was conducted to assist with urination until the establishment of a reflex to empty the bladder. A behavior test was performed daily for each hindlimb using the BBB scale. Two trained investigators who were blind to the experimental conditions performed the behavioral analyses. After modeling, all groups were randomly divided into 3 subgroups corresponding to different time points after SCI (1 day, 3 days, and 7 days), with 3 rats for each time point. All surgeries were performed by the same person (S Sohn). The sham group did not receive any treatment. The TUDCA group received an intraperitoneal injection of 200 mg/kg of TUDCA immediately after the injury. That dose was repeated 24 h after the injury. The injury group received the same dose of saline intraperitoneally at the same time points.

### Hematoxylin & Eosin (H&E), Luxol Fast Blue/cresyl violet, and Immunofluorescent Staining

At appropriate time points, nine rats from each group were given an overdose of anesthesia. After cannulation of the left ventricle-ascending aorta, rapid perfusion with ice-cold saline was performed. When the efflux became clear, the rats were perfused using 4% paraformaldehyde/phosphate buffered saline (PBS) for 5 minutes (in 200 ml). The spinal cords were exposed from the original incisions in the back. Centered at the injury site, a segment of the spinal cord approximately 1.5 cm in length was dissected, fixed overnight in 4% paraformaldehyde/PBS, dehydrated in 15% sucrose for 1 day followed by 30% sucrose for 2–3 days. After treatment with the sucrose solutions, the tissues were embedded in OCT compound (Sakura, Tokyo, Japan) and frozen at −80 °C. Axial serial sections with a slice thickness of 16 μm were generated and six serial sections were randomly selected from each tissue block and stained with a Hematoxylin & Eosin (H&E) solution (BBC biochemical, Mount Vernon, USA) and luxol fast blue/cresyl violet-staining. According to standard procedures for immunofluorescent staining, cryosections were treated with a blocking solution to prevent a nonspecific binding reaction for 1 h and then stained by incubation overnight at 4 °C with the following primary antibodies in PBS: monoclonal mouse anti-mouse CD68 (1:100; ab955; Abcam, Cambridge, UK), and polyclonal anti-rabbit iNOS (1:100; ab15323; Abcam) or monoclonal anti-mouse CD86 (1:100; ab213044; Abcam) and polyclonal anti-goat Arg-1 (1:100; ab92274; Abcam). The slides were then incubated with fluorescent secondary donkey anti-mouse Alexa 488, and donkey anti-rabbit Alexa 568 in a blocking solution or anti-goat Alexa 647 (final dilution, 1:400; Invitrogen) for 1 hour at room temperature. At the end, nuclei were stained with DAPI and sections were washed in PBS and mounted with a specific medium (DakoCytomation, Milan, Italy). After H&E staining and immunofluorescent staining, fields were selected using the most severely injured spinal cord segment as the center, and the morphological changes of the H&E staining and luxol fast blue/cresyl violet-staining samples were observed under a light microscope. The fluorescent expression of immunofluorescent staining was determined using a confocal laser-scanning microscope (TCS SP5; Leica Microsystem Microscope, Wetzlar, Germany).

### Statistical analyses

All values are presented as the mean ± standard deviation (SD). Behavioral scores were analyzed by unpaired Student’s t-test. A one-way analysis of variance (ANOVA) with subsequent post-hoc tests were used to verify statistically significant differences among the groups. Differences with p-values for which *p < 0.05, **p < 0.01, and ***p < 0.001 were considered to be statistically significant.
